# A Sensorized 3D-Printed Knee Test Rig for Preliminary Experimental Validation of Patellar Tracking and Contact Simulation

**DOI:** 10.3390/s24103042

**Published:** 2024-05-10

**Authors:** Florian Michaud, Francisco Mouzo, Daniel Dopico, Javier Cuadrado

**Affiliations:** Laboratory of Mechanical Engineering, Centro de Investigación en Tecnologías Navales e Industriales (CITENI), Campus Industrial de Ferrol, University of La Coruña, 15403 Ferrol, Spain; francisco.mouzo@udc.es (F.M.); daniel.dopico@udc.es (D.D.); javier.cuadrado@udc.es (J.C.)

**Keywords:** experimental validation, musculoskeletal modeling, contact simulation, knee simulator, multibody dynamics, contact forces, patellar tracking, 3D printing, orthopedic surgery, motion capture

## Abstract

Experimental validation of computational simulations is important because it provides empirical evidence to verify the accuracy and reliability of the simulated results. This validation ensures that the simulation accurately represents real-world phenomena, increasing confidence in the model’s predictive capabilities and its applicability to practical scenarios. The use of musculoskeletal models in orthopedic surgery allows for objective prediction of postoperative function and optimization of results for each patient. To ensure that simulations are trustworthy and can be used for predictive purposes, comparing simulation results with experimental data is crucial. Although progress has been made in obtaining 3D bone geometry and estimating contact forces, validation of these predictions has been limited due to the lack of direct in vivo measurements and the economic and ethical constraints associated with available alternatives. In this study, an existing commercial surgical training station was transformed into a sensorized test bench to replicate a knee subject to a total knee replacement. The original knee inserts of the training station were replaced with personalized 3D-printed bones incorporating their corresponding implants, and multiple sensors with their respective supports were added. The recorded movement of the patella was used in combination with the forces recorded by the pressure sensor and the load cells, to validate the results obtained from the simulation, which was performed by means of a multibody dynamics formulation implemented in a custom-developed library. The utilization of 3D-printed models and sensors facilitated cost-effective and replicable experimental validation of computational simulations, thereby advancing orthopedic surgery while circumventing ethical concerns.

## 1. Introduction

Despite the continuous advances in implant design and surgical techniques, numerous Total Knee Replacement (TKR) complications are still observed, with 10% of them being associated with patellar complications, which may require the repetition of surgical procedures [1]. To avoid extensor mechanism complications and ensure good functional outcomes, obtaining proper patellar tracking is one of the most important goals of TKR. Poor patellar tracking can result in increased postoperative contact pressures, patellar tilt, patella subluxation, or dislocation [2]. The use of musculoskeletal models in orthopedic surgeries offers significant potential to mitigate surgical complications through the prediction of post-treatment function. Computational simulations empower clinicians to assess diverse treatment options, diminish subjectivity in treatment planning, and enhance clinical outcomes for individual patients [3,4,5,6,7]. These virtual models and experiments emulate real-world phenomena using mathematical algorithms and computer software, facilitating the exploration, comprehension, and prediction of the behavior of complex systems or processes that may be difficult, costly, unsafe, or not ethical to study directly [8,9]. However, despite the significant promises of this method, the research community has only made limited progress in validating its predictions due to the scarcity of direct in vivo measurements [10], which are limited for technological and ethical reasons. Having experimental data not only allows researchers to assess the model’s fidelity and reliability but also enables them to fine-tune simulation parameters, thereby avoiding the introduction of errors external to their mathematical approach. Ensuring that simulations are trustworthy and accurately represent real-world phenomena is essential for surgeons and the scientific community, particularly when considering their predictive capabilities.

Developing methods and algorithms to accurately model the complex interactions of contacting bodies in simulations poses significant challenges. When dealing with colliding bodies with intricate 3D geometries, a comprehensive collision detection algorithm becomes necessary [4,11]. To simulate the behavior of the patellofemoral joint, two primary approaches have been reported in the literature: multibody dynamics (MBD) [4,5,12,13,14] and the Finite Element Method (FEM) [15,16,17]. Nevertheless, when acquiring experimental data for parameter tuning and result validation, the authors employed various strategies. Some utilized magnetic resonance images at different static knee angles [15,17], while others utilized dynamic computed tomography scans [13], and some used cadaveric knees [14]. All these methods imply ethical restrictions and access to clinical resources, which can delay, limit, or prohibit a substantial part of the research community when trying to generate significant new advances in the field. Additionally, the use of cadaveric data to derive generic model information also poses challenges related to scalability, practical applicability limits, and cost [18,19]. Given the limitations mentioned above, some researchers opted to validate their results by comparing them with experimental data reported in the literature [4,12,16]. However, these validations were limited to agreement with envelope measurements and do not guarantee an accurate representation of subject-specific behavior. Finally, it is worth mentioning that despite several attempts to estimate patellar contact pressure and motion, none of these studies obtained experimental measurements of pressure. Only Elias et al. [13] obtained motion data through dynamic computed tomography scans, which present the side effects of radiation exposure. 

In this study, to streamline the validation process of their novel mathematical algorithm for simulating the patellar trajectory post-TKR, the authors have developed a sensorized 3D-printed knee test rig. This low-cost and replicable solution enabled them to conduct preliminary experimental validation of their findings while circumventing ethical concerns through the use of 3D-printed models and sensors. This simplified validation process boosts confidence in the model’s predictive abilities and its suitability for real-world applications, serving as an initial step in the development project before significant time and resources are invested. For this purpose, a commercial training station for knee ligament release that recreates a human leg [20] was adapted. This tool consists of articulated metal supports for the hip and foot and replaceable inserts for the knee joint. In order to obtain a virtual replica of the system, the geometries of the implants were virtually applied, and the resulting cut bones were 3D printed, with real tibial and femoral implants being placed in the respective physical bone models. In addition, an innovative sensorized patella has been proposed to experimentally measure its complete motion and the forces applied to it. Specifically, a prosthetic patellar button was affixed to a pressure sensor using 3D-printed supports, onto which three optical markers were attached. The patella was linked to the tibia through a spring and to the femur via another spring with lower stiffness. The springs represent the patellar tendon and the quadriceps tendon, respectively, and were connected in series to respective load cells to measure their tensions. The movements of the femur, tibia, and patella were captured using an optical motion capture system. The various experimental data recorded served two purposes: firstly, as inputs and parameter adjustments for the simulation, and secondly, the recorded patellar movements, in conjunction with the forces registered by the pressure sensor and load cells, were used to validate the simulation results obtained from a multibody dynamics formulation implemented in a custom-developed library [11,21]. 

## 2. Material and Methods

### 2.1. Test Bench

**Figure 1:**
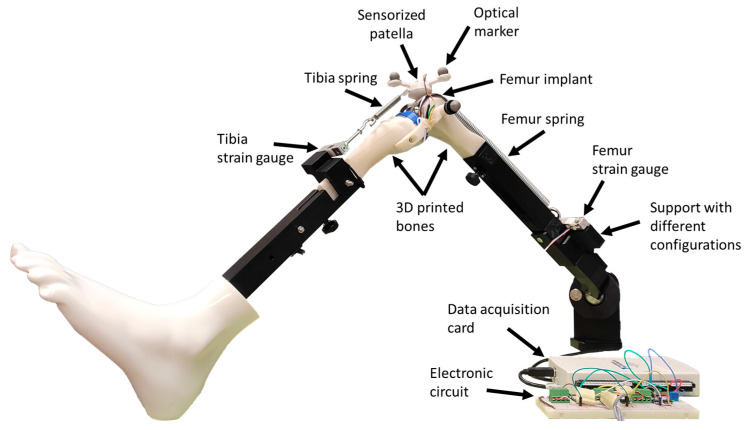
Sensorized test bench.

The commercial training station (Mita Collateral Ligament Release Workstation, Bristol, UK) for knee ligament release [20], shown in Figure 1, was adapted for this work. The current commercial structure, which replicates a human leg (consisting of a base with a spherical joint for the hip, black metal supports for bones, and a polypropylene foot), was chosen for its convenience and aesthetic appeal. However, any mechanical system, whether commercial or homemade, equipped with a spherical joint could serve the same purpose. The original knee inserts were replaced with personalized 3D-printed bones incorporating their corresponding implants, and springs and multiple sensors with their respective supports were added. In addition, an innovative sensorized patella has been proposed to experimentally measure its complete motion and the forces applied to it.

The 3D-printing process begins with obtaining customized CAD models of the patient’s bones from medical images. These models can then be used to simulate the effects of various treatments virtually. Additionally, 3D printing enables the production of physical models of these bones with the applied treatment, such as cuts in this case. As a result, two corresponding models are generated: the digital model and the physical model. In this work, the case of TKR was addressed. Therefore, the virtual geometries of the implants were applied, and the resulting cut bones were 3D printed (Prusa I3 MK3S, Prague, Czech Republic), with commercial implants (Microport^®^, Shanghai, China) of the tibia and femur being placed in their respective bone models.

Motion and force sensors enable the reproduction of movement in the virtual model, adjustment of simulation parameters, and experimental validation of the results. The movements of the femur, tibia, and patella were recorded by an optical motion capture system. Six optical markers were placed to capture the movement of the three bodies constituting the system (two additional markers were used to determine the hip center, which was fixed, through a calibration capture). Due to the preliminary nature of this work, the cruciate ligaments were released (as it happens in the surgery), and the collateral ligaments were treated as rigid bodies (to avoid contact between femur and tibia implants), allowing only one degree of freedom at the knee, in addition to the three rotations at the hip. The patella, on the other hand, was considered a fully free body, in contact with the femoral implant, attached to the tibia via one spring (Figure 1, tibia spring), and to the femur via another spring with lower stiffness (Figure 1, femur spring). To recreate different patellar tracking, the 3D-printed support of the femur strain gauge allowed two different configurations. 

**Figure 2:**
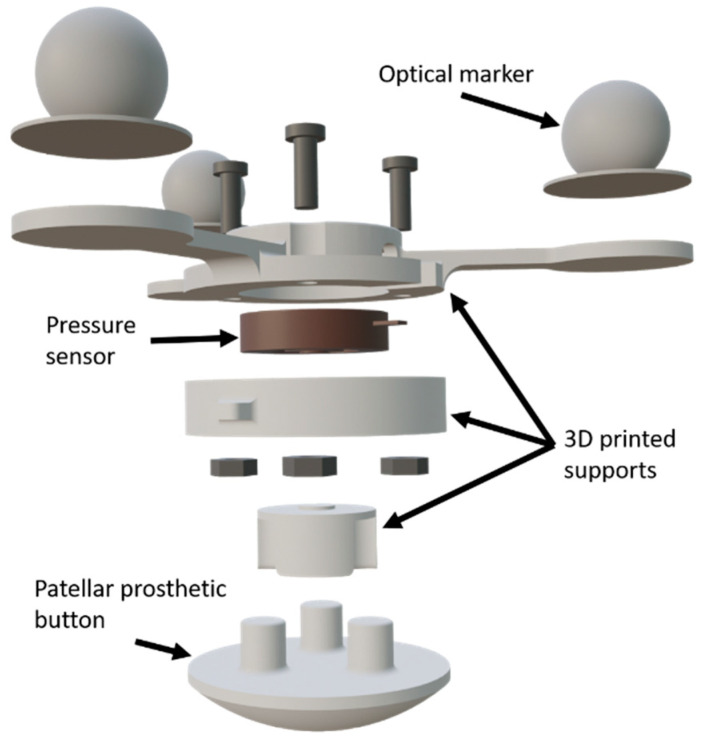
Sensorized patella.

The springs represent the patellar tendon and the quadriceps tendon, respectively, and were connected in series with load cells to measure their tensions (Figure 1). For the patella, as seen in Figure 2, a prosthetic patellar button was attached to a pressure sensor through 3D-printed supports onto which three optical markers were fixed. In order to mimic the lubricating effect of synovial fluid in the joint, lubricant was applied to the contacting surfaces.

### 2.2. Movement and Experimental Data Collection

The patellar trajectory is defined as the movement of the kneecap relative to the femoral groove during knee flexion and extension [22]. Traditionally, assessment of the patellar trajectory is subjectively performed by the surgeon during the operation, relying on direct visualization [23]. After placing the implants in the corresponding bones, the surgeon manually flexes and extends the knee of the anesthetized patient, to observe the range of motion of the joint and evaluate the patellar trajectory after the applied treatment. During this routine maneuver, the surgeon looks for any signs of lateral subluxation (movement of the patella to the outside of the knee) or malalignment. While the motion may appear simple because the patient lies on his back, without muscle activity due to anesthesia, this method was shown to be relevant for assessing patellar tracking during TKR.

**Figure 3:**
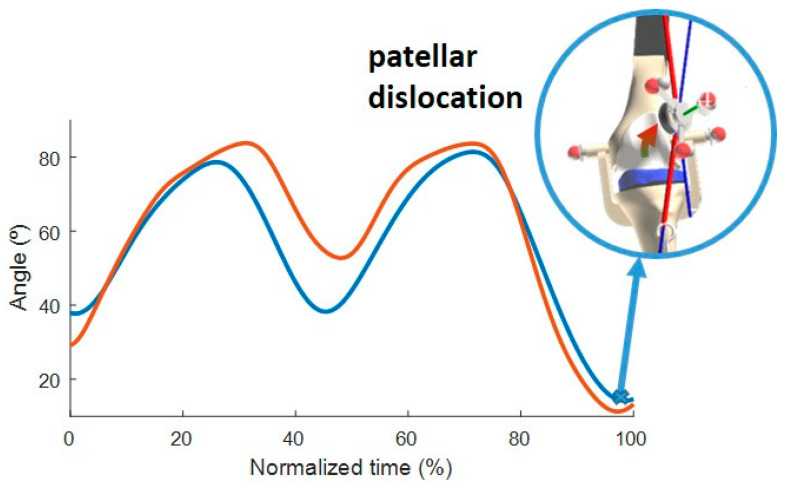
Knee angle during motion (blue: configuration A; orange: configuration B).

In this study, the maneuver has been replicated and two manual knee flexions and extensions (Figure 3) were performed to observe the patellar trajectory. The position of the optical markers was recorded using 18 infrared cameras (OptiTrack FLEX 3, Natural Point, Corvallis, OR, USA) at a sampling frequency of 100 Hz. Additionally, spring tensions were recorded using two tension load cells (RB-Phi-119, Phidgets, Calgary, AB, Canada), and the pressure of the prosthetic button on the femur was measured using a compact pressure load cell (FX29, TE Connectivity, Wört, Germany), also at a sampling frequency of 100 Hz. A second-order Butterworth filter with a cutoff frequency of 12 Hz was applied to the optically captured marker trajectories [24], and a singular spectrum analysis (SSA) [25] with a window length of 30 was applied to the force measurements.

To validate different configurations, two distinct trajectories of the patella were measured by modifying the attachment point of the spring to the femur using the adjustable support (Figure 1). This modification corresponds to altering the Q angle, also known as the quadriceps angle, which measures the alignment of the quadriceps muscles and the patella relative to the femur [26]. In this case, configuration A was laterally displaced by 20 mm compared to configuration B, resulting in a 4.55° difference between the respective Q angles. In configuration A, poor patellar tracking with patellar dislocation was generated when the knee flexion was lower than 20° (Figure 3). As shown in Figure 3, the motion started with the leg flexed around 35° and was then flexed until 90°, extended to 45°, flexed again to 90°, and, finally, extended until 10°, thus resulting in a patellar dislocation for configuration A but not for configuration B. Due to the manually executed experimental actuation, the imposed motion was not exactly the same for both configurations.

### 2.3. Computational Model

**Figure 4:**
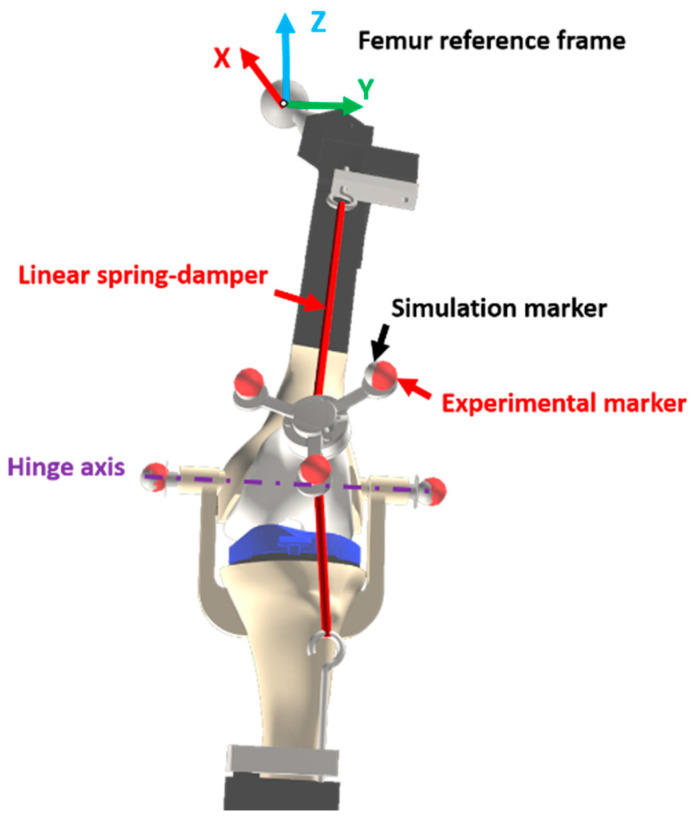
Computational model.

The leg model considered in this work consisted of three rigid bodies: the femur, the tibia-foot assembly, and the patella. The 3D geometries were identical to the physical pieces, both for the supports and the bones and implants. While the femur was fixed at the hip joint and could rotate in three directions, the joint between the femur and tibia was modeled as a hinge around the mechanical axis identified by two optical markers located on the sides of the knee (Figure 4, in purple). Thus, both the real leg mechanism and the virtual one had four degrees of freedom: three at the hip and one at the knee. The patella, on the other hand, was a free body with six degrees of freedom, interacting with the leg through two linear springs/dampers (Figure 4, red lines: one attached to the femur and the other to the tibia) and the contact with the femoral implant.

The geometrical and physical parameters of the rigid bodies (local coordinates of points, masses, inertias, etc.) were estimated from CAD models created in Solidworks and introduced, along with the mechanical constraints of the system, into a custom-developed library [21]. The mechanical parameters of the springs (stiffness, natural length, and damping) were estimated from the experimentally recorded positions and forces during calibration.

### 2.4. Simulation

Several studies using FEM have investigated the patellofemoral joint [15,16,17]. However, despite its potential for managing knee osteoarthritis, the time-intensive nature of FEM, including pre-processing, processing, and post-processing, limits its clinical applicability [27]. Considering this limitation and the expectation of minimal elastic deformations in the relevant bones, the investigation of the MBD approach arose as a feasible solution [4,5,12]. The simulation algorithm was written in Fortran 2008 and C++ and implemented in a custom-developed library [11,21]. This method offers a computationally efficient approach to address clinical concerns related to the knee joint. 

#### 2.4.1. Guiding

The positions and orientations of the rigid bodies were obtained from the recorded marker positions captured by the cameras. To achieve this, the traditional approach described by Vaughan [28] was used, which consists of the following steps: (i) selecting three non-collinear entities, which can be markers or already located joints, within each segment; (ii) defining an orthogonal reference frame for the corresponding segment based on the three selected entities; (iii) using correlation equations to estimate the position and orientation of the rigid body [24].

The movements of the femur and tibia recorded by the motion capture optical system were used as inputs for the simulation. Therefore, the four free angles of the leg (three at the hip and one at the knee) were guided with the experimental values. The recorded movements of the patella served to experimentally validate the simulation results (Figure 4, red markers) as well as to approximate the patella to its initial static equilibrium position, which had to be determined.

#### 2.4.2. Formulation

The selected formulation for the multibody system dynamics in this work was the ALI3-P formulation, explained in [29], which has been developed over many years as an evolution of the formulations presented in [30,31]. It is an Augmented Lagrangian formulation of index 3 in mixed coordinates (natural plus relative), with velocity and acceleration projections on the constraint subspaces. 

The configuration of the multibody system was described by a set of *n_c_*-dependent coordinates $q\in R^{n_{c}}$. These coordinates were related through a set of *m* holonomic constraint equations Φ(q,t)=0, and the equations of motion were expressed in the following manner:

(1a) $${\left[M\ddot{q}\right]}_{\delta m}+{\left[\Phi_{q}^{T}\lambda^{*(i+1)}+\Phi_{q}^{T}\alpha \Phi \right]}_{\delta f}=Q_{\delta f},$$

(1b) $$\lambda_{n+1}^{*(i+1)}=\lambda_{n+1}^{*(i)}+\alpha \Phi_{n+1}^{\left(i+1\right)};\;\;i>0,$$

with **M = M(q)** and $Q=Q(q,\dot{q})$ being the mass matrix and generalized force vector, respectively, $\Phi_{q}$ the Jacobian matrix of the constraints vector, **λ** the Lagrange multipliers vector, **α** a diagonal matrix that contains the penalty factors associated with the constraints, *δf* and *δm* scalar parameters of the generalized-α method, *n* the time step index, and *i* the iteration index of the approximate Lagrange multipliers, $\lambda_{n+1}^{*}$. For a complete description of the formulation of the equations of motion and projections of velocities and accelerations, the reader is referred to [11].

The integration scheme adopted was the Newmark integrator [32], with a time-step size of 1 ms.

#### 2.4.3. Static Equilibrium

To perform a dynamics simulation of a multibody system, it is necessary to obtain a set of initial positions and velocities that satisfy the constraint equations, both at position and velocity levels. Additionally, in those multibody systems that have a defined static equilibrium position, it is advisable to start the simulation from the static equilibrium position to avoid the presence of initial high accelerations that could disturb the stability of the simulation. This involves solving the static equilibrium equations of the system to determine the equilibrium position. Unfortunately, when the system involves bodies in contact, solving the static equilibrium problem becomes very complex, and there may even be multiple solutions.

In this work, the static equilibrium equations were obtained by eliminating the accelerations and velocity-dependent forces in the equations of motion, that is, by solving the following equations:

(2a) $$[\Phi_{q}^{T}\lambda^{*(i+1)}+\Phi_{q}^{T}\alpha \Phi {]}_{0}=Q_{0},$$

(2b) $$\lambda_{0}^{*(i+1)}=\lambda_{0}^{*(i)}+\alpha \Phi_{0}^{\left(i+1\right)};i>0,$$

where *i* = 0, 1, 2, …, and the subscript 0 in the expressions of (1) indicates that quantities are evaluated at the initial time.

For solving the nonlinear system (1), a Newton–Raphson iteration was used, similar to the one used to solve the equations of motion [11].

#### 2.4.4. Contact Model and Detection

Since the contact area had been lubricated in the test bench, the approach proposed in this work to address the contact problem between the patella and the femoral implant considers only the normal forces and not the tangential forces (friction). The chosen normal force model for this work was the Flores model [33], and the expression for the normal force has the following form:

(3) $$F_{n}=k_{n}\delta^{p}\left(1+\frac{8(1-\varepsilon )}{5\varepsilon }\frac{\dot{\delta }}{{\dot{\delta }}_{0}}\right)n,$$

where $k_{n}$ is the equivalent stiffness of the contact, which depends on the shape and the material properties of the colliding bodies, *p* is the Hertz’s exponent, δ is the indentation and $\dot{\delta }$ its temporal derivative, ${\dot{\delta }}_{0}$ is the relative normal velocity between the colliding bodies when the contact is detected, ε is the coefficient of restitution, and **n** is the direction of the force. The subscript “**n**” comes from “normal”.

Since the colliding bodies had complex 3D geometries, a general collision detection algorithm was required. While a (triangular) mesh-to-mesh contact algorithm was used in previous works [11,34], to check the penetration between bodies and find the corresponding contact points, in this work, an algorithm based on the analytic formulation of the 3D geometry of the patellar prosthetic button was preferred [35].

**Figure 5:**
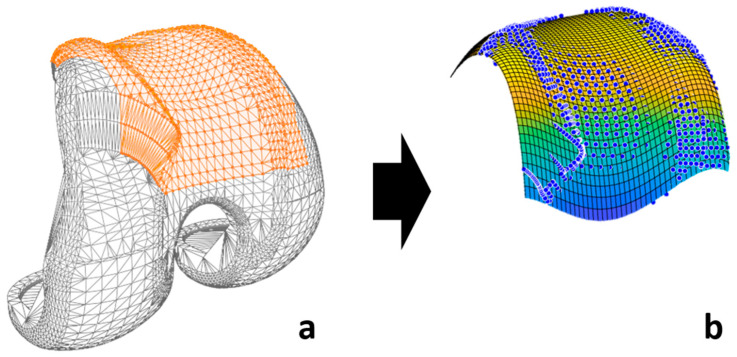
(**a**) Contact surface considered (orange); (**b**) analytical representation of the 3D geometry of the femoral implant (blue dots: original surface).

Analytic formulation involves using mathematical equations to calculate the distances between the surfaces of the geometric primitives. The specific equations used depend on the types of primitives involved. In this work, only the contact between the patellar button and the femur implant was considered. A sphere primitive was employed for the patellar button, since only its spherical portion would come into contact with the femur. A similar simplification was applied to the femur implant geometry, focusing solely on the surface that would interact with the patellar component (highlighted in orange in Figure 5a). Subsequently, this complex surface was approximated using a custom-made Matlab program by means of a 4th-order polynomial equation from the vertex coordinates with an R-squared value of 99.2% (Figure 5b).

**Figure 6:**
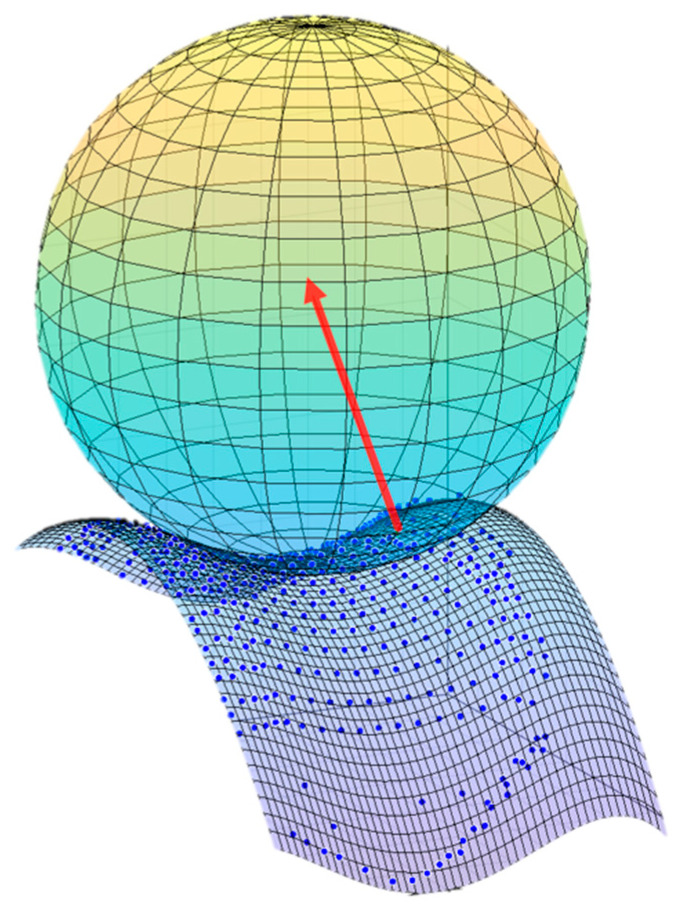
Representation of the normal contact force (red arrow) between the patellar prosthetic button (green) and the femoral implant (blue dots: original surface, blue surface: analytical representation).

These equations take into account the position, orientation, and size of the primitives. The simulation needs to determine whether or not two objects are in contact at a certain time point. If the distance between the center of the sphere (patellar button) and the femur surface is smaller than the sphere radius, a collision or contact exists. Based on this information, and for each detected contact, the normal force is computed (perpendicular to the contact surfaces, Figure 6, in red) using the aforementioned contact model. The collision detection algorithm and the contact force model were implemented in the in-house developed library [21]. 

#### 2.4.5. Experimental Validation

In order to validate the results obtained from the computational simulations, the recorded experimental measurements were used as a reference. The forces applied on the patella were validated by comparing the forces of the springs with the measurements from the load cells, and the contact force (only the normal component) with that obtained from the pressure sensor. The position of the patella during the motion was also validated by comparing the coordinates of the center of the patellar prosthetic button with the coordinates recorded by the optical motion capture system, both with respect to the femur reference frame to avoid error accumulation (Figure 4). The anatomical coordinate system of the femur was defined as follows: the origin was the hip center, the Y-axis was defined by the two markers located on each side of the knee, and the Z-axis was defined by the vector normal to the Y-axis and the vector between the hip and knee centers. The error was quantified for the two configurations (A and B) by conducting a Bland–Altman analysis between pairs of datasets. 

#### 2.4.6. Computational Details

All the analyses were run on an Intel (R) Core (TM) i7-13700KF @ 3.40 GHz, 32 GB RAM, SSD 2TB with a Windows 10 Pro operating system. The single-threaded program was written in Fortran 2008 and C++ and compiled with MSVC 2017 and Intel Fortran 2018. The MA28 suit was used as the linear algebra package. To measure efficiency, the run-time was chosen, defined as the time required to solve the initial static equilibrium and run the simulation.

#### 2.4.7. Optimization

For both configurations, the results were first obtained using the spring parameters derived from calibration measurements. Subsequently, an optimization process was performed to enhance the results and observe their sensitivity to these parameters. The spring parameters were allowed to vary by 10% from their default values, and the objective function was defined as the sum of the RMSE of the forces (contact and springs) and the distance error of the relative position of the patella. The minimum value of the function was estimated in Matlab using the genetic algorithm (*ga* function).

## 3. Results

### 3.1. Sensorized 3D-Printed Knee Test Rig and Sensorized Patella

To obtain a preliminary validation of their new mathematical algorithm for simulating post-TKR patellar trajectory, the authors proposed a sensor-equipped 3D-printed knee test rig. This cost-effective and reproducible solution allowed them to conduct, in the first weeks of the project, the experimental validation of their ideas by means of 3D-printed models and sensors, while avoiding ethical concerns. The recorded experimental data had a dual role: first, they enabled the reproduction of movement in the virtual model and the adjustment of the numerous simulation parameters; second, the recorded movements of the innovative sensorized patella, along with the forces measured by the pressure sensor and load cells, were utilized to validate the simulation results. The total cost of the sensorized system, including the commercial training station, was under EUR 5000 (motion capture system and 3D printer excluded). This initial validation procedure enhanced confidence in the model’s predictive capacity and its relevance to practical settings, marking the outset of the development project before substantial time investments were made. 

### 3.2. Computational Time

In the case of the described simulation as a validation example, the run-times required to simulate the 3.64 s of real motion of configuration A and the 5.00 s of real motion of configuration B were, respectively, 1.42 s and 1.67 s. These values are, approximately, 2.77 times faster than real time.

### 3.3. Comparison with Experimental Data

**Figure 7:**
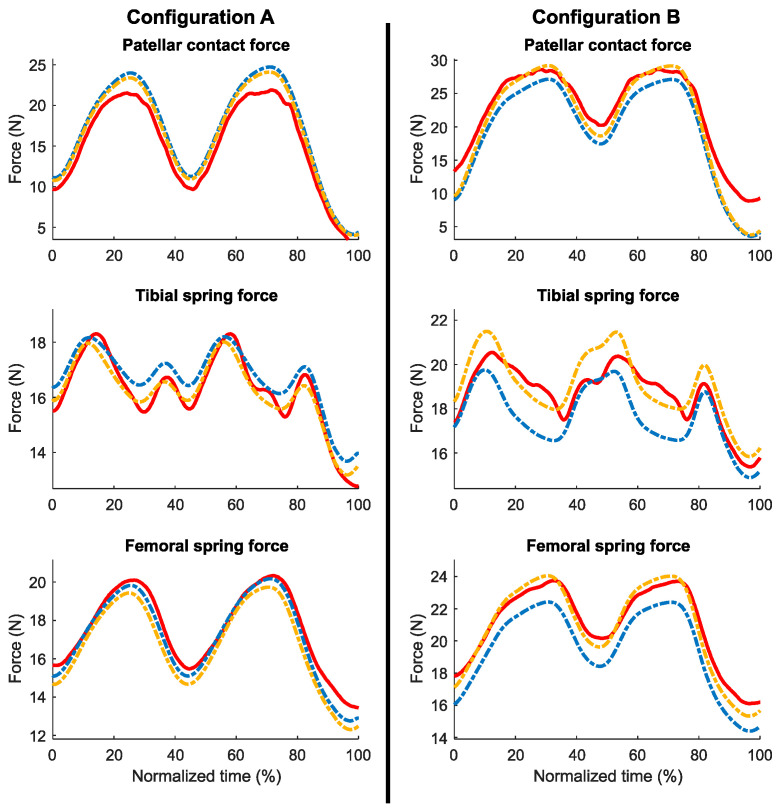
Comparison between experimental (red) and simulation forces (blue: original; yellow: optimized).

The results indicate that the forces obtained through computational simulation (Figure 7; blue: original; yellow: optimized) followed a similar pattern to the forces obtained from experimental measurements (Figure 7, red). Despite the calibration, the forces from the springs at the initial instant (slightly flexed knee) were approximated with a small error (less than 1.5 N error for configuration A and 4.2 N for configuration B, without optimization). During knee flexion, the forces of the springs increased and generated more pressure on the patella. However, the tibial spring force did not increase throughout the complete flexion. After a certain angle of knee flexion, both configurations showed a reduction in spring length and force. This behavior was accurately replicated by the simulation.

**Figure 8:**
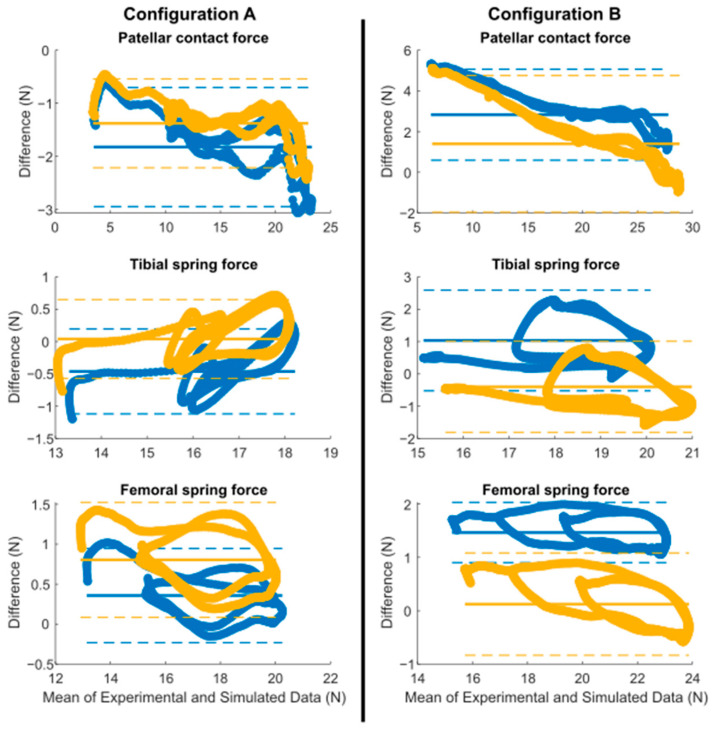
Bland–Altman analysis between experimental and simulation forces (blue: original; yellow: optimized; dot: data; line: bias; dashed line: limits of agreement).

The peak values of the forces show a proportional error to the initial offset, since it was maintained throughout the motion. The Bland–Altman analysis uncovered biases among the various datasets (Figure 8 and Table 1). It was noted that the errors were predominantly systematic, and the optimization process played a significant role in reducing them (highlighted in yellow). The values were primarily located within their limits of agreement, represented by dashed lines. The greatest error was noted for the contact force in configuration B, with biases of 2.8 N and 1.4 N before and after optimization, respectively. In terms of the overall simulation, the forces were estimated with high accuracy. The mean bias of all forces was below 1.8 N with the default parameters and below 0.8 N after optimization (Table 1).

**Figure 9:**
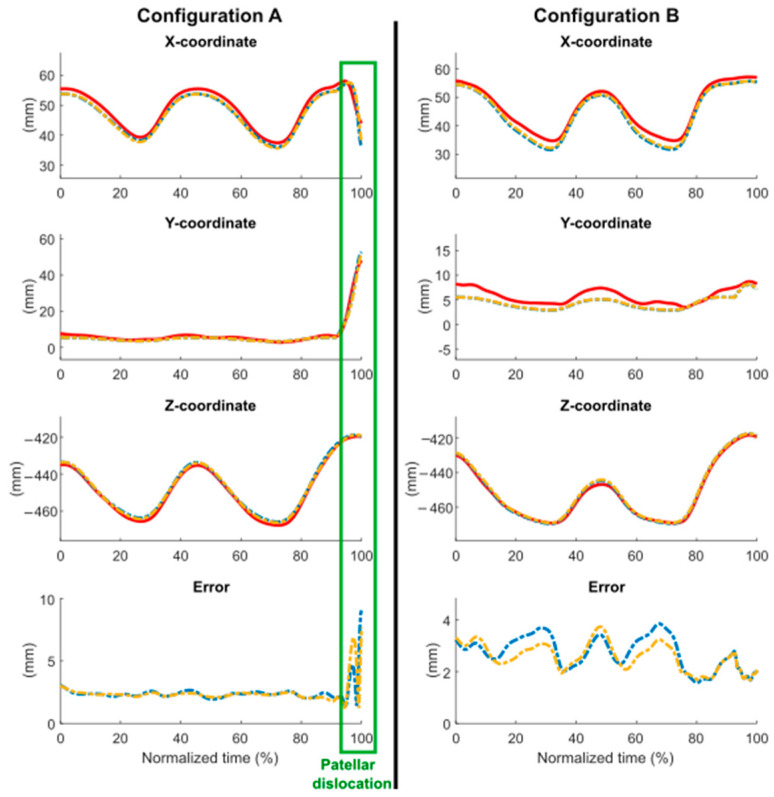
Comparison of experimental (red) and computational patellar positions (blue: original, yellow: optimized).

**Figure 10:**
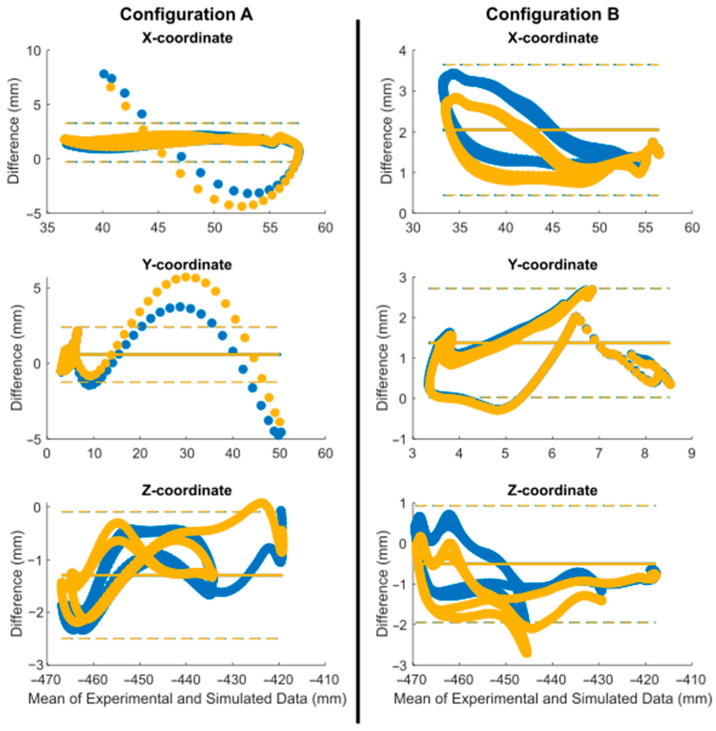
Bland–Altman analysis between experimental and computational patellar positions (blue: original; yellow: optimized; dot: data; line: bias; dashed line: limits of agreement).

The simulated movement of the patella also showed very small discrepancies with respect to the motion recorded by the optical motion capture system (Figure 8). It was observed that the patella started its movement with positional errors of 3.1 mm and 3.2 mm for configurations A and B, respectively, resulting in discrepancies in all three coordinates of the studied point. The Bland–Altman analysis revealed discrepancies among the different datasets (Figure 9 and Figure 10 and Table 1). It was observed that the errors exhibited a systematic pattern, and the optimization process notably contributed to mitigating them (highlighted in yellow). The values were primarily within their limits of agreement, depicted by dashed lines, except for the instances of patellar dislocation (configuration A), which exhibited higher errors during the patella’s displacement out of the femur. The average error incurred during the complete motion was lower than 2.8 mm for the distance and lower than 2.1 mm for the coordinates, consistent across all the simulations. The maximum bias was reduced to 1.65 mm thanks to the optimization process. Patellar dislocation was successfully reproduced during the simulation of configuration A, both with the default and the optimized values of the parameters.

## 4. Discussion

### 4.1. Sensorized 3D-Printed Knee Test Rig and Sensorized Patella

In this study, a commercial surgical training station was transformed into a sensorized test bench to provide an affordable solution for preliminary experimental validation that can be replicated by research groups without access to extensive financial, experimental, and clinical resources. The use of 3D-printed models and sensors allowed low-cost and reproducible experimental validation of computational simulation (patellar movement and forces) while avoiding ethical issues, which can delay, limit, or prohibit a substantial part of the research community when trying to generate significant new advances in the field. 

It is important to note that the simulations of this work involve challenging contact modeling and detection using a complex multibody dynamics formulation implemented in a custom-developed library. During the process, before adjusting all the parameters, the simulations yielded reasonable results, which could have been considered acceptable. However, upon comparison with experimental data from the real world, it became evident that these initial results were far from reality and needed improvement. This improvement was made possible only by using the personalized sensorized knee rig. 

The cost and complexity of the proposed system are very affordable compared to knee simulators developed by universities such as Oxford, Kansas, and Purdue [36,37]. Additionally, the total cost could be halved by utilizing simpler mechanical supports instead of the commercial training station, which was selected for our convenience and for its aesthetic appeal. This development provides an affordable solution for experimental validation that can be replicated by research groups with limited financial, experimental, and clinical resources. 

Furthermore, in this work, a novel fully sensorized patella has been designed by incorporating a pressure sensor, which has the potential to lead to the development of new instrumented implants or to improvements in existing knee simulators. Despite several attempts to estimate patellar contact pressure and motion, none of these studies obtained experimental measurements of pressure. Only Elias et al. [13] obtained motion data through dynamic computed tomography scans, which present the side effects of radiation exposure.

### 4.2. Computational Time

The obtained computational times are promising, making the novel approach suitable for running optimizations to determine anatomical or treatment parameters and for conducting intraoperative simulations. None of the works studying the patellofemoral joint with FEM have reported computational times [15,17]. However, the time-intensive nature of FEM is well-known, thus limiting its clinical applicability [27]. Among the studies that utilized MBD, only Bei and Fregly [12] reported calculation times. Their simulations of contact between the femur and tibia required 1 min of CPU time, and their dynamic simulations took 10 min. However, it is important to note that these results were obtained twenty years ago.

### 4.3. Comparison with Experimental Data

Although the system was simplified for this preliminary study, the mechanical behavior exhibited was quite complex due to the contacts between bodies, resulting in a complex solution for the initial static equilibrium position, including the existence of multiple equilibrium positions [11]. The obtained results showed that the computationally simulated forces followed patterns similar to those of the experimentally measured forces, but small biases were maintained throughout the motion. These differences could be the result of small discrepancies in the equilibrium position and slight discrepancies in the spring parameters. Nevertheless, force predictions were primarily within the limits of agreement of the Bland–Altman analysis, and the optimization of the spring parameters significantly reduced the biases. The overestimation of patellar contact forces in configuration A could be attributed to an underestimation of the measurements, caused by the actuation of non-centered force pressures on the sensor. 

Small biases were also observed in the position of the patella compared to the position recorded by the optical motion capture system. When attempting to position the patella at the experimentally recorded initial position, it was observed that the 3D geometries of the patella and femoral implants were not in contact but rather had a small separation of a few millimeters. This could be due to defects in 3D printing, inaccuracies in optical measurements exacerbated by their processing and the body motion reconstruction from them, or inaccuracies in the analytical approximation of the geometry of the femoral implant. While the optical motion capture system is considered a reference in terms of precision [24,38], it is quite reasonable to expect errors of a couple of millimeters within a capture volume of more than 25 m^3^ with markers of 14 mm in diameter. Kwak et al. [14] reported positional errors and obtained average errors of around 1 mm. Nonetheless, they only compared static poses and utilized an accurate but expensive three-dimensional coordinate measuring machine model CX-D2. 

Despite the small discrepancies, which are common between the real and virtual worlds, the obtained results were very satisfactory and allowed validation of the models used in the study. The observed patellar dislocation in configuration A along the second extension was successfully reproduced, even when using the default values of spring parameters. These results will also pave the way for other implementations (e.g., for other treatments) and will be communicated to healthcare professionals.

### 4.4. Limitations

The authors acknowledge that the current study relies on several assumptions and simplifications in modeling the knee joint, which may introduce inaccuracies and limitations in the simulation results. Consequently, these preliminary findings may be currently limited in their applicability to real human knees.

Before attempting to predict treatments, the objective of this preliminary study was to demonstrate the ability to faithfully replicate the surgeon’s maneuver, which has clinical relevance. While this common maneuver may seem simple, it is sensitive enough to detect signs of lateral subluxation (movement of the patella to the outside of the knee) or malalignment [23]. It has been demonstrated that, by making minor adjustments to the Q angle, a patellar dislocation can be observed. This simplified validation procedure instills greater trust in the model’s predictive accuracy and its relevance to real-world situations, providing an initial checkpoint in the development process before committing extensive resources. In addition, it is worth mentioning that this case study is particularly sensitive to small biases because only the spring forces are active during passive knee flexion. Applying muscle forces will likely render these discrepancies insignificant due to their higher magnitude.

The authors acknowledge that the knee joint is more complex than a simple revolute joint. However, this complexity is not relevant to the patellar trajectory studied in this work, which is defined as the movement of the kneecap relative to the femoral groove during knee flexion and extension. Since their novel mathematical approach aims to simulate the patellar contact forces, and the motion of the tibia and femur was controlled in this work, the joint linking them becomes irrelevant. Even if the tibia was defined as an independent body, it would only affect the position of the attached point of the spring in the experimental motion capture and the simulation, and this study demonstrated that changes resulting from the modification of the attachment points of the springs were accurately reproduced. Additionally, it must be noted that the surgeon’s maneuver, as well as the replicated experimental measurement, involves manual actuation. This makes it very difficult to apply the corresponding forces to the virtual model since the force magnitude and the application points/surfaces are not evident. This issue needs to be resolved, especially if the contact between femoral and tibial implants is added to the problem for a more comprehensive treatment prediction.

### 4.5. Future Works

In the subsequent phase of simulation development, the authors progressively enhanced the system’s intricacies to more accurately represent anatomy. These modifications included replacing the simplified hinge knee joint with springs on both sides, representing the lateral ligaments, and incorporating the direct contact between the tibia and femur, among other aspects. This tool allowed for the comparison of accuracy among various collision detection algorithms used in simulating contact between patellar and femoral implants, with a focus on high-performance computing [39]. These initial validation steps contributed to strengthening the confidence in the model’s predictive accuracy and its suitability for real-world scenarios, marking the inception of the development project prior to substantial investments of time and resources. 

In future endeavors, we plan to conduct a sensitivity analysis to evaluate the effects of alterations in system parameters and enhance the stability and reliability of the simulations. As additional improvements, we are considering reconstructing the model’s motion during activities such as walking or squatting, solving inverse dynamics, and estimating contact forces, all while offering real-time visualizations of the results [40].

## 5. Conclusions

The authors have presented a low-cost and reproducible knee simulator that avoids ethical issues by utilizing 3D-printed models and sensors. This streamlined validation process enhances trust in the model’s predictive accuracy, serving as an initial validation checkpoint prior to a significant investment of time and resources. Their model serves as a valuable tool for validating patellar tracking and contact simulation post-TKR, offering a cost-effective solution accessible to research groups with restricted financial, experimental, and clinical resources. With the aid of recorded motion and force sensors, the system allows the recreation of virtual movements, fine-tuning of numerous simulation parameters, and validation of experimental outcomes. Furthermore, this study introduces an innovative sensorized patella design, featuring a pressure sensor integration.

## Figures and Tables

**Table 1:** Biases with respect to experimental data.

		Configuration A		Configuration B	
		Original	Optimized	Original	Optimized
Bias	Contact Force (N)	−1.82	−1.38	2.83	1.40
	Tibial Spring Force (N)	−0.46	0.04	1.03	−0.40
	Femoral Spring Force (N)	0.36	0.80	1.47	0.12
	X-coord. (mm)	1.51	1.58	2.04	1.65
	Y-coord. (mm)	0.59	0.72	1.37	1.33
	Z-coord. (mm)	−1.29	−1.09	−0.51	−1.03

## Data Availability

The datasets generated for this study are available on request to the corresponding author.
